# Association Between Trigger Surprisal and Tension-Type Headache Attacks

**DOI:** 10.18103/mra.v13i9.6969

**Published:** 2025-10-01

**Authors:** Dana P. Turner, Twinkle Patel, Timothy T. Houle

**Affiliations:** 1 Department of Anesthesia, Critical Care and Pain Medicine Massachusetts General Hospital, Harvard Medical School, Boston, MA, USA; 2 Department of Neurology, UMass, Worcester, MA, USA

## Abstract

**Background:**

The causes of individual headache attacks are commonly sought, yet the multiple potential influences make this task difficult. Information theory provides a framework for addressing this challenge by quantifying how unexpected an exposure is through *surprisal*. Prior research has shown that higher surprisal scores predict migraine onset, but the extent to which this relationship generalizes to tension-type headache remains unknown.

**Aims:**

This study aimed to determine whether surprisal is associated with incident tension-type headache attacks among individuals with episodic migraine.

**Methods:**

This secondary analysis proceeded from a prospective daily diary study in which 109 participants with migraine recorded potential triggers, headache activity, and symptoms twice daily for up to 28 days. Surprisal values were computed from person-specific probability distributions of diary responses, aggregated to yield average surprisal scores per diary entry. Associations between current surprisal and the onset of headache attacks within 12- and 24-hour intervals were evaluated. Analyses were conducted for all headaches combined and separately for migraine-only and tension-type headache-only attack sets.

**Results:**

Headache attacks occurred in 1,345 of 4,530 (29.7%) of 12-hour and 2,122 of 4,947 (42.9%) of 24-hour windows. Stratified analyses showed a strong association for migraine attacks, OR: 2.18 (95%CI: 1.15 – 4.14) at 12 hours and OR: 2.88 (95%CI: 1.77 – 4.69) at 24 hours. In contrast, associations with tension-type headache were weak and nonsignificant, OR: 1.01 (95%CI: 0.45 – 2.23) at 12 hours and OR: 1.40 (95%CI: 0.69 −2.86) at 24 hours. Exploratory nonlinear and contextual analyses within the tension-type headache subset revealed no consistent gradients or effect modification by prior-day surprisal.

**Conclusions:**

Surprisal was associated with migraine but not tension-type headache attacks in this cohort. These findings suggest that migraine may be more sensitive to contextual unpredictability in the environment than tension-type headache. Future research should examine surprisal in populations with primary tension-type headache diagnoses to clarify whether the absence of association reflects true diagnostic differences or misclassification of attacks.

## Introduction

Many individuals with headache attempt to determine the causes, or “triggers,” of their headache attacks.^1^ Often, these individuals compare changes in presumed trigger exposure with occurrence of headache attacks.^2,3^ In essence, this covariation assessment requires an individual to consider what exposure, or pattern of exposures, is atypical based on experience. Presumably, because headache attacks are rare for most individuals, attention should be paid to trigger exposures that are also rare in occurrence. However, because there are a great number of potential headache triggers,^4^ and the effect of any trigger may be delayed, this endeavor is very complicated. Thus, it is extremely difficult to isolate the effects of a specific trigger factor.^5,6^

A helpful way to conceptualize the broad range of migraine triggers is through the lens of information theory,^7^ which links the information conveyed by a variable to the uncertainty in its occurrence.^8^ Information theory is closely linked to statistical inference and has been used to describe the amount of information in a host of variables from a wide array of fields. Within this framework, the information contained in a single trigger exposure can be quantified as its *surprisal*, or the degree of unexpectedness associated with observing that trigger.^9^ Events that are rare or unexpected carry more information than those that are common or anticipated. For binary triggers (present vs. absent), surprisal is calculated as −log₂(p), where *p* is the probability of the event, yielding values in bits of information. Higher amounts of information (i.e., more bits) imply more uncertainty in the pattern of exposure to a variable, like a headache trigger. Applying measures such as surprisal provides a unified metric for representing a wide variety of potential headache triggers on a common scale, offering a promising foundation for developing a universal system of headache trigger assessment.^9^

Indeed, prior research has shown that rare or unexpected values of common headache triggers, such as caffeine and alcohol consumption, stress, and mood disturbances, are consistently linked to increased headache attack risk.^10^ Furthermore, aggregating individual trigger surprisals into a total surprisal score yielded stronger discrimination between headache and non-headache days than considering any single trigger in isolation. A subsequent replication study considered an even greater number of triggers while examining the association between daily surprisal scores and the onset of episodic migraine attacks within 12- and 24-hour intervals.^11^ Analyses of longitudinal data using person-specific statistical models demonstrated that elevated surprisal scores were reliably associated with an increased likelihood of future headache attacks. These findings confirm earlier results in a new, prospectively collected dataset and highlight surprisal as a promising metric for forecasting migraine risk.

However, several challenges may limit the practical use of a surprisal-based measurement system. A key issue is how to estimate the likelihood of an event before extensive data have been collected. Traditional surprisal calculations rely on well-sampled empirical distributions, but in applied clinical settings, the available data may be limited. This raises the question of how to judge the surprisal of an event without weeks of prior observations. Previous studies have relied on retrospective data,^10,11^ where surprisal was calculated only after distributions were established. For surprisal to be useful in forecasting migraine risk or modeling real-time brain–environment interactions, it must be possible to approximate event likelihood under limited data conditions. We have explored prospective Bayesian approaches for estimating surprisal in low-data contexts, moving from a retrospective analytic tool to a prospective predictive framework.^12^ A second challenge is the need to track a wide range of potential headache influences that also requires considerable work to measure a vast array of variables. To reduce participant burden and simplify measurement procedures, we have explored the notion of experience sampling and item-response theory to support practical implementation.^13^

To expand on the previous work that has examined surprisal measurements in only headache attacks from individuals with episodic migraine, it would be worthwhile to explore the association between surprisal and other types of headache attacks. The objective of this paper is to examine how surprisal methods work for headache attacks that are not migrainous in nature. We hypothesize that the surprisal metric will be associated with incident tension-like headache attacks similar to those of migraine.

## Methods

This is a secondary analysis of these data. Four pre-planned primary analyses have been previously published.^9,11–13^ These data were collected through a longitudinal daily diary study after Institutional Review Board approval. Participants, recruited through local advertisements (i.e., institutional online recruitment system, public transportation, flyers) were enrolled for up to 28 days during the period of April 2021 to December 2024. To be eligible for participation, individuals completed a telephone screening and must have been 18 to 65 years old and must have been experiencing 4 to 14 headache days per month with an International Classification of Headache Disorders, 3rd Edition migraine diagnosis with or without aura.^9^ Exclusion criteria included a secondary headache disorder, medication over-use headache or chronic daily headache, a change in the nature of their headache symptoms during the last 6 weeks, inability to read or speak English at the 6^th^ grade level, an unmanaged Axis I psychotic disorder, substance dependence (e.g., alcohol, marijuana) that would interfere with headache activity and data collection, and pregnancy or anticipated pregnancy during the study period.

Those who met eligibility criteria and agreed to participate completed the informed consent process before beginning a series of questionnaires in an in-person or virtual enrollment session. These questionnaires included demographic information, Migraine Disability Assessment (MIDAS),^14^ and headache characteristics and were completed electronically using REDCap software.^15^ Participants were then taught how to complete the at-home twice-daily diaries. These diaries were also conducted in REDCap and involved completing a 5–10 minute entry each morning and evening. After 28 days of participation, individuals completed a final in-person or virtual questionnaire session.

### Daily Diaries

The electronic diaries included questions about possible headache triggers, headache activity, and use of medications. Each diary entry also included questions on the nature of any headache attack that had occurred since the previous entry (e.g., tension-type headache and migraine symptoms). Further details of individual items are described elsewhere.^9^ Briefly, the morning (AM) diaries included sleep-related questions such as bed time, wake time, quality, duration, and awakenings. They also included the Profile of Mood States Short Form (POMS-SF),^16^ weather, and late-night eating. The evening (PM) diaries included the POMS-SF, Daily Stress Inventory (DSI),^17^ common food and drink triggers, meal patterns and missed meals, weather, and environmental exposures.

### Surprisal System

The concept of surprisal was used to quantify the unexpected nature of a participant’s daily response patterns.^7,9,10^ Surprisal was defined as the negative logarithm of the probability of an observed exposure (−log_2_ [p_exposure_]), representing how unlikely an event is under a given probability distribution. For this study, individual-specific probability distributions were generated from each participant’s observed responses over the study period. Within each diary entry (AM and PM), surprisal values were calculated for each item based on these person-specific distributions. Diary total surprisal was then obtained by summing the item-level values and dividing by the number of completed items, producing an average surprisal per item. This scaling approach accounts for missing data and allows AM and PM diaries to be placed on a comparable scale, despite the PM entries containing more items. The resulting metric provides a within-person index of how atypical a day’s responses are relative to that individual’s usual patterns.

### Outcomes

The primary outcome was the presence or absence of a self-reported headache attack of any pain intensity (i.e., > 0 on a 0 to 10 scale). Individual attacks were further categorized as being either migraine or tension-type by their characteristics and secondary symptoms using the International Classification of Headache Disorders (ICHD-3).^18^ For analysis, two future time horizons, 12 hours and 24 hours were a priori defined based on their relation to a diary entry. If two attacks were reported in any 24-hour period, they were classified as migraine if either attack met ICHD criteria for migraine.

### Statistical Power Considerations

The statistical power considerations have been previously reported.^9,11^ In brief, the sample size was designed to allow precision of estimates around observed event rates related to the definition of the surprising events. There were no a priori considerations made for this analysis, where the association between surprisal and future attacks is further subdivided based on diagnosis. Thus, the analysis proceeded based on the available sample size and was not explicitly designed to detect clinically meaningful associations for any diagnostic subgroups. To allow interpretation in the context of potentially low power, effect estimates are provided with 95%CI.

### Statistical Analyses

All analyses were conducted in R 4.5.1 and R-Studio (2025.05.1+513). Descriptive statistics were used to summarize sample characteristics, frequency of attacks, and the distribution of attack types across participants and time horizons. Median [25^th^, 75^th^] were computed for continuous variables, while frequencies and percentages were used for categorical variables. To estimate the association between current surprisal and future headache attacks, mixed-effects logistic regressions were fit with a logit link using maximum likelihood. Separately for the 12-hour and 24-hour outcomes, the probability of any future headache was modeled as a function of concurrent headache status (present or absent), current surprisal (bits), and diary type (e.g., AM/PM), with a participant-specific random intercept and random slope for the scaled surprisal score. These models were repeated in each subtype-restricted analytic set: (i) migraine-only, defined as diaries where any subsequent headache (if present) was classified as migraine (vs no headache), and (ii) tension-type–only, defined analogously for tension-type headache. Two-sided tests were used throughout, with statistical significance defined as *p* < 0.05. Fixed-effect estimates are presented as odds ratios with 95% confidence intervals and *p*-values.

In Turner et al.,^11^ non-linear associations based on lagged surprisal relationships were observed using these data. Thus, we also conducted contextual (effect modification) analyses within the tension-type–only sets to evaluate whether prior-day surprisal (surprisal lag) modified associations between current surprisal and future headache. Final contextual models are presented, and their marginal effects are visualized by plotting predicted probabilities over the scaled surprisal scores at representative surprisal lag values (e.g., 0.3, 0.6, 0.8 bits).

## Results

Participant characteristics have been described previously.^9,11^ In brief, 109 individuals with migraine were enrolled (median age 35 years [26.0–46.0]); most were female (102/109, 93.5%) and White (91/109, 83.5%). Of those enrolled, 104 completed twice-daily electronic diaries for up to 28 days, contributing 5,176 total entries. Across the cohort, the median monthly headache frequency was 8 days [5.0–12.0], and the median peak intensity was 7/10 [5.5–8.0]. Headaches were commonly unilateral (79/109, 72.5%) and pulsating (57/109, 52.3%), with frequent associated symptoms of photophobia (105/109, 96.3%), phonophobia (95/109, 89.6%), and nausea/vomiting (99/109, 90.8%). The median MIDAS score was 24 [13.0–35.5], indicating moderate to severe disability. See Table 1 for more details.

### Association Between Surprisal and Each Headache Type

Headache occurred in 29.7% (1,345/4,530) of observations within 12 hours and 42.9% (2,122/4,947) within 24 hours in the all-headache set using complete cases with all information provided for each variable in the model. In the migraine-only set, headache frequency was 21.8% (890/4,075) and 33.8% (1,442/4,267) and was 12.2% (442/3,627) and 19.4% (678/3,503) for 12 and 24 hours, respectively, in the tension-type set. Thirteen attacks at 12 hours and two attacks at 24 hours could not be classified due to missing secondary symptom information (See Table 2).

**Figure 1.**
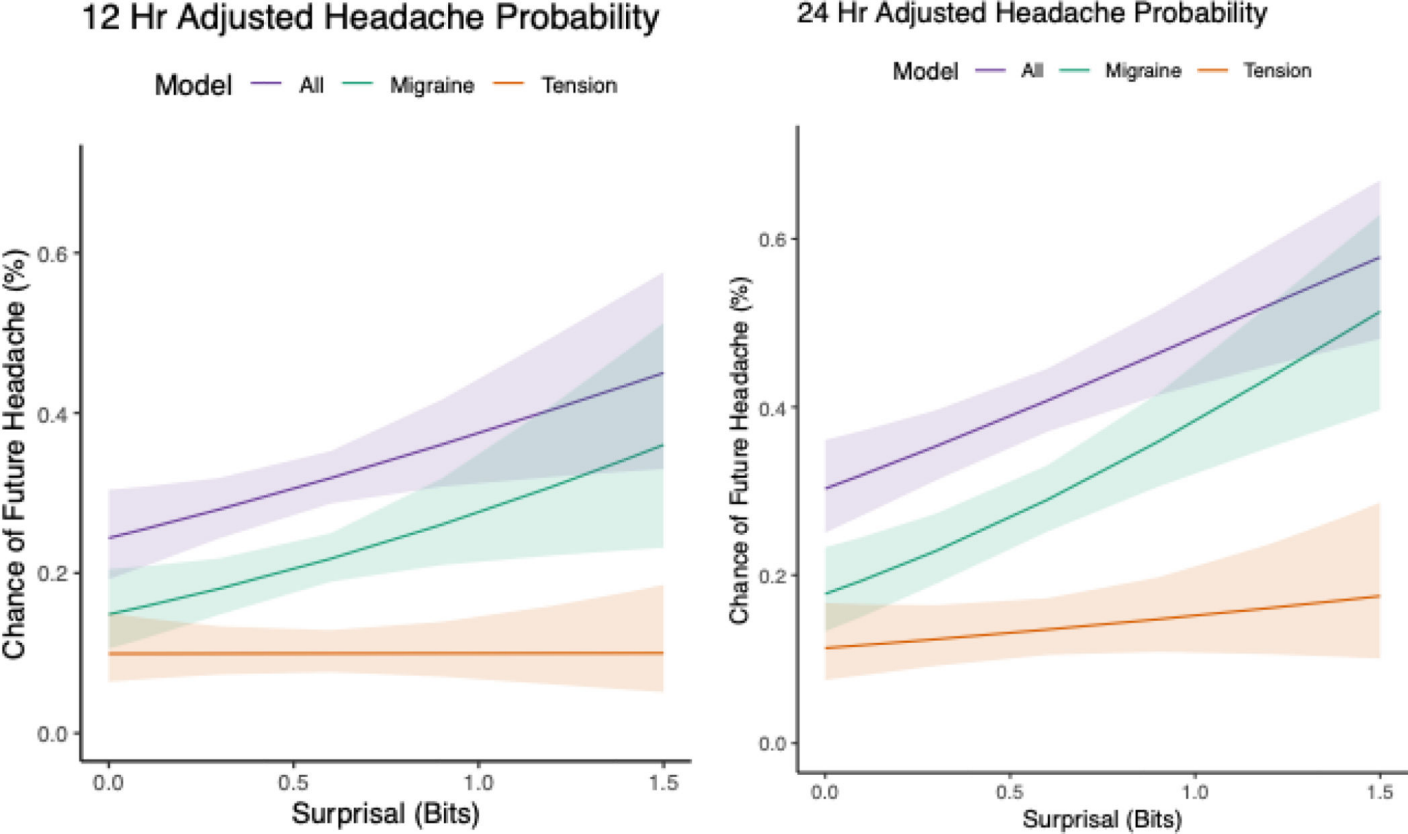
Predicted probability of future headache within 12-hour (left) and 24-hour (right) windows as a function of current surprisal. Higher surprisal was strongly associated with migraine attacks but showed little to no relationship with tension-type headache.

Higher surprisal was associated with greater odds of a future headache attack of any kind, OR: 1.86 (95%CI: 1.12–3.08), p=0.016 at 12 hours and OR: 2.15 (95%CI: 1.44–3.20), p<0.001 at 24 hours (previously reported^11^). However, stratifying the analysis by types of attack revealed that the association was strong for migraine attacks, OR: 2.18 (95%CI: 1.15–4.14), p=0.017 at 12 hours and OR: 2.88 (95%CI: 1.77–4.69), p<0.001 at 24 hours, but this was not evident or was weak for tension-type headache, OR: 1.01 (95%CI: 0.45–2.23), p=0.990 at 12 hours and OR: 1.40 (95%CI: 0.69–2.86), p=0.351 at 24 hours. Figure 1 displays the strength of the association for each type of headache.

### Exploring Contextual and Non-Linear Associations for Tension-Type Headache

**Figure 2.**
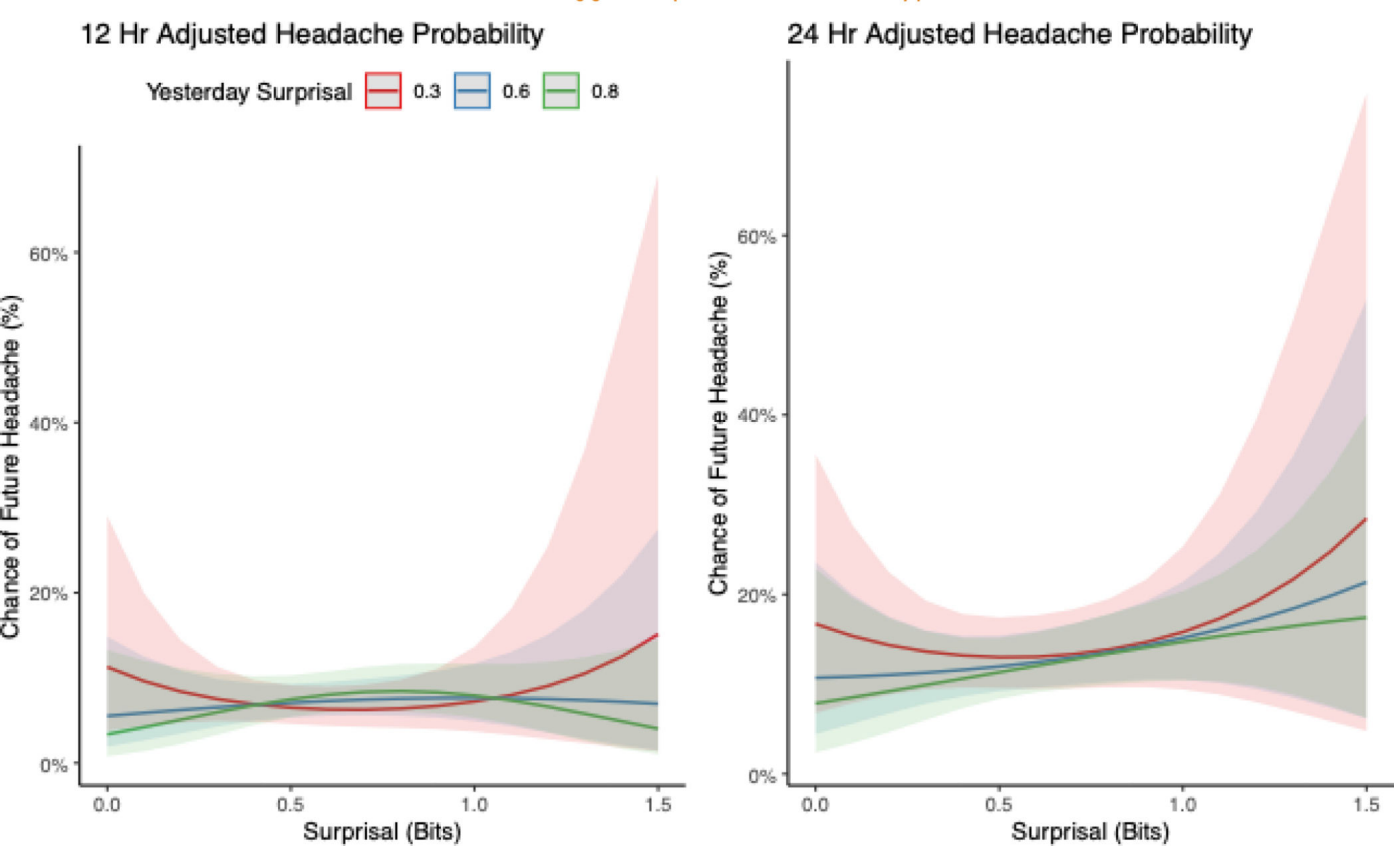
Predicted probability of future tension-type headache within 12-hour (left) and 24-hour (right) windows as a function of current surprisal, stratified by recent surprisal values. Although the association was stronger for 24 hours, no consistent association or effect modification was observed across the combination of surprisal levels.

Within the tension-type headache subset, nonlinear and contextual analyses indicated little to no enhanced relationship between current surprisal and subsequent headache at either 12 or 24 hours. Predicted risks remained low and largely flat across the surprisal range, with minimal separation among curves stratified by prior-day surprisal (0.3, 0.6, 0.8). Any increases at higher surprisal values were small and imprecise, with wide, overlapping confidence bands. Although the association was stronger over the 24-hour horizon than the 12-hour horizon, no consistent gradient by current surprisal, or modification by prior-day surprisal, was evident. Overall, these findings suggest that associations in the tension-type subset are weak or absent (See Figure 2).

## Discussion

This secondary analysis extends previous work demonstrating that higher surprisal, a dynamic, entropy-based metric reflecting deviation from an individual’s expected daily experience, is associated with increased risk of headache onset.^11^ The current study aimed to evaluate whether this relationship was specific to migraine attacks or if it generalized to tension-type headache attacks in individuals with episodic migraine. As previously reported, higher surprisal was associated with a greater likelihood of future headache attacks overall.^11^ However, stratified analyses revealed that the association was strong and consistent for migraine attacks but weak or absent for tension-type headache attacks, regardless of the prediction window (12 or 24 hours).

This diagnosis-specific pattern may suggest that surprisal is more closely aligned with the neurobiological and behavioral dynamics of migraine than with those of tension-type headache. Migraine is known to be preceded by premonitory symptoms^19^ and may be more sensitive to internal and external disruptions, features which surprisal may implicitly capture. In contrast, the flatter and less predictive risk profiles observed for tension-type headache imply that the mechanisms driving these headaches may be less influenced by abrupt contextual shifts or deviations from expectation. These findings support the clinical and conceptual distinction between migraine and tension-type headache and suggest that surprisal may be a useful prognostic tool for migraine but not for tension-type headache.

Conversely, research on headache triggers demonstrates considerable overlap between migraine and tension-type headache triggers.^19^ In both conditions, individuals frequently attribute headache onset to common factors such as stress, sleep disturbance, fatigue, missed meals, hormonal changes, and certain environmental stimuli (e.g., weather changes, bright light, noise).^20^ Studies that directly compare migraine with tension-type headache show that most triggers are shared, though the prevalence or strength of association may differ.^21^ It is important to note that there is a distinction between assessing the belief systems from individuals with headache versus examining the actual association between trigger exposures and incident headache attacks.^3^ While most research on headache triggers utilizes surveys to characterize individual beliefs about what causes attacks,^4^ in the current study the actual association was evaluated independently of individual beliefs.

The interpretation of these findings is limited by several important factors. First, although attacks were classified based on ICHD-3 criteria, all participants in the sample were individuals with episodic migraine, and thus the observed tension-type headache attacks may not reflect the full clinical profile of individuals with a primary tension-type headache disorder. It is plausible that these attacks represent less severe or atypical manifestations of migraine rather than true tension-type headache events. Second, the classification of tension-type headache attacks was imperfect. Attacks labeled as tension-type headache in this study were operationally defined as merely those failing to meet criteria for migraine rather than being independently validated as tension-type headache. Some of these episodes may have been treated early, suppressing the development of migraine-associated features (e.g., photophobia, phonophobia), leading to misclassification. Third, the sample was not powered to detect associations within diagnostic subgroups. The analyses were conducted post hoc and were exploratory in nature. Thus, the absence of a strong signal in the tension-type headache group should be interpreted cautiously, as small or moderate associations may have gone undetected. Effect estimates are presented with confidence intervals to reflect this uncertainty.

Future research should prioritize the inclusion of individuals with a primary clinical diagnosis of tension-type headache to more accurately characterize the surprisal-tension-type headache relationship. The current study’s tension-type headache sample was derived from a migraine-enriched sample, potentially limiting its generalizability to true tension-type headache populations. Prospective studies enrolling participants with confirmed episodic or chronic tension-type headache as their primary headache disorder are essential to determine whether surprisal or similar entropy-based constructs hold predictive value in this clinical phenotype. Such work should consider alternative symptom constellations, temporal patterns, and contextual drivers that may be more relevant for tension-type headache than those observed in migraine. Additionally, integrating ecological momentary assessment with physiological or behavioral markers may enhance model performance and yield insights into the often underexplored prodromal phase of tension-type headache.

Attention should also be paid to whether the application of a surprisal scoring system can enhance headache self-management, independent of headache diagnosis. By quantifying deviations from expected daily experiences, surprisal scores may help individuals recognize and appreciate the complex array of influences on their health, thereby increasing their sense of internal locus of control and self-efficacy.^22–24^ A stronger internal locus of control has been associated with improved coping, reduced disability, and better outcomes in patients with chronic pain and headache disorders.^22^ Moreover, digital self-tracking approaches in migraine and other conditions have shown that greater awareness of triggers and early warning signals can empower individuals to engage in adaptive self-regulation.^25,26^ By monitoring surprisal, patients could be guided toward personalized management strategies that not only prevent or preempt attacks through early and effective medication management but also prepare them with tailored coping responses. For example, an emerging literature indicates that efforts to avoid specific headache triggers may actually be counterproductive (see: ^27,28^). By condensing the combined impact of multiple environmental and behavioral factors into a single surprisal score, individuals can better appreciate that fluctuations in experience are unavoidable and that effective management requires coping rather than avoidance. Such approaches may ultimately support resilience, reduce headache burden, and improve quality of life across all headache phenotypes.

## Conclusions

In conclusion, the previously reported associations between surprisal and future headache were reaffirmed for migraine attacks, but no evidence was found to support a similar relationship in tension-type headache attacks. While these findings are consistent with the hypothesis that migraine is more responsive to contextual unpredictability, the results cannot confirm a differential effect, as all analyses were conducted within the same dataset used to establish the primary surprisal–migraine association. The apparent absence of an association in tension-type headache may reflect true diagnostic differences, limitations in attack classification, or insufficient statistical power. Future studies should enroll individuals with primary tension-type headache diagnoses and evaluate surprisal-based forecasting in larger, diagnostically diverse samples to determine whether these findings represent a genuine lack of predictive utility or an artifact of measurement and design.

## Figures and Tables

**Table 1.** Participant and Headache Characteristics (N = 109)*

**Participant Characteristics**		
Age	35	[26.0, 46.0]
Sex		
Male (%)	7	(6.5%)
Female	102	(93.5%)
Race (%)		
American Indian or Alaska Native	3	2.8%
Asian	9	8.3%
Black	6	5.5%
White	91	83.5%
Hispanic (%)	11	10.2%
Marital Status (%)		
Divorced	1	0.9%
Married	44	40.7%
Separated	2	1.9%
Single	59	54.6%
Widowed	2	1.9%
**Headache Characteristics**		
Headache Frequency	8	[5.0, 12.0]
Multiple Headache Types	63	57.8%
Headache Intensity	7	[5.5, 8.0]
Location		
Unilateral	79	72.5%
Bilateral	29	26.9%
Pulsating Quality	57	52.3%
Nausea or Vomiting		
Never	10	9.2%
>= Sometimes	99	90.8%
Photophobia		
Never	4	3.7%
>= Sometimes	105	96.3%
Phonophobia		
Never	11	10.4%
>= Sometimes	95	89.6%
Aggravated by activity		
Never	8	7.5%
Sometimes	99	90.8%
Visual Aura	43	39.4%
MIDAS Total	24	[13.0, 35.5]

Values are frequency counts (%) or median [25^th^, 75^th^]

* Reprinted with permission from Turner et al. (2025)^9^

**Table 2.** Association Between Surprisal and Future Headache by Headache Type

	12 hours		24 hours	
Model	Headache/N Total (%)	OR (95%CI) p-value	Headache/N Total (%)	OR (95%CI) p-value
All Headaches*	1345 / 4530 (29.7%)	1.86 (1.12 – 3.08) 0.016	2122/4947 (42.9%)	2.15 (1.44 – 3.20) < 0.001
Migraine Only	890/4075 (21.8%)	2.18 (1.15 – 4.14) 0.017	1442/4267 (33.8%)	2.88 (1.77 – 4.69) < 0.001
Tension-Type Headache Only	442/3627 (12.2%)	1.01 (0.45 – 2.23) 0.990	678/3503 (19.4%)	1.40 (0.69 – 2.86) 0.351

* Reported in Turner et al. (2025)^11^

All models adjusted for diary type (^AM^, PM) and current headache status
